# Emergency department direct discharge compared to short-stay unit admission for selected patients with acute heart failure: analysis of short-term outcomes

**DOI:** 10.1007/s11739-023-03197-9

**Published:** 2023-02-21

**Authors:** Carolina Sánchez-Marcos, Javier Jacob, Pere Llorens, María Pilar López-Díez, Javier Millán, Francisco Javier Martín-Sánchez, Josep Tost, Alfons Aguirre, María Ángeles Juan, José Manuel Garrido, Rafael Calvo Rodríguez, Enrique Pérez-Llantada, Elena Díaz, José Andrés Sánchez-Nicolás, María Mir, Esther Rodríguez-Adrada, Pablo Herrero, Víctor Gil, Alex Roset, Frank Peacock, Òscar Miró, The ICASEMES Research Group researchers

**Affiliations:** 1 Digital Cultures & Societies, University of Queensland, Mianjin/Brisbane, Spain; 2 Emergency Department, Hospital Universitari de Bellvitge, L’Hospitalet de Llobregat, Barcelona, Catalonia Spain; 3 Emergency Department, Instituto de Investigación Sanitaria Y Biómedica de Alicante (ISABIAL), Short Stay Unit and Hospital at Home, Hospital General de Alicante, Miguel Hernández University, Alicante, Spain; 4 Emergency Department, Hospital Universitario de Burgos, Burgos, Spain; 5 Emergency Department, Hospital Universitario La Fe, Valencia, Spain; 6 Emergency Department, Hospital Clínico San Carlos, Universidad Complutense, Madrid, Spain; 7 Emergency Department, Consorci Hospitalari de Terrassa, Barcelona, Catalonia Spain; 8 Emergency Department, Hospital del Mar, Barcelona, Catalonia Spain; 9 Emergency Department, Hospital Dr. Peset, Valencia, Spain; 10 Emergency Department, Hospital Virgen de La Macarena, Seville, Spain; 11 Emergency Department, Hospital Reina Sofía, Córdoba, Spain; 12 Emergency Department, Hospital Marqués de Valdecilla, Santander, Spain; 13 Emergency Department, Hospital Sant Joan, Alicante, Spain; 14 Emergency Department, Hospital Reina Sofía, Murcia, Spain; 15 Emergency Department, Hospital Rey Juan Carlos, Móstoles, Madrid, Spain; 16 Emergency Department, Hospital Infanta Leonor, Madrid, Spain; 17 Emergency Department, Hospital Central Asturias, Oviedo, Spain; 18 Emergency Department, Baylor College of Medicine, Houston, TX USA

**Keywords:** Acute heart failure, Death, Mortality, Emergency department, Revisit, Hospitalization

## Abstract

Short stay unit (SSU) is an alternative to conventional hospitalization in patients with acute heart failure (AHF), but the prognosis is not known compared to direct discharge from the emergency department (ED). To determine whether direct discharge from the ED of patients diagnosed with AHF is associated with early adverse outcomes versus hospitalization in SSU. Endpoints, defined as 30-day all-cause mortality or post-discharge adverse events, were evaluated in patients diagnosed with AHF in 17 Spanish EDs with an SSU, and compared by ED discharge vs. SSU hospitalization. Endpoint risk was adjusted for baseline and AHF episode characteristics and in patients matched by propensity score (PS) for SSU hospitalization. Overall, 2358 patients were discharged home and 2003 were hospitalized in SSUs. Discharged patients were younger, more frequently men, with fewer comorbidities, had better baseline status, less infection, rapid atrial fibrillation and hypertensive emergency as the AHF trigger, and had a lower severity of AHF episode. While their 30-day mortality rate was lower than in patients hospitalized in SSU (4.4% vs. 8.1%, *p* < 0.001), 30-day post-discharge adverse events were similar (27.2% vs. 28.4%, *p* = 0.599). After adjustment, there were no differences in the 30-day risk of mortality of discharged patients (adjusted HR 0.846, 95% CI 0.637–1.107) or adverse events (1.035, 0.914–1.173). In 337 pairs of PS-matched patients, there were no differences in mortality or risk of adverse event between patients directly discharged or admitted to an SSU (0.753, 0.409–1.397; and 0.858, 0.645–1.142; respectively). Direct ED discharge of patients diagnosed with AHF provides similar outcomes compared to patients with similar characteristics and hospitalized in a SSU.

## Introduction

Heart failure (HF) is an important health problem with elevated socio-health care costs. It is highly prevalent in people over 65 years of age and constitutes the most common first cause of hospitalization in this population. In addition, mortality and rehospitalization associated with decompensations of acute heart failure (AHF) are high, even in patients with low-risk HF [1–3]. The emergency department (ED) plays a key role in management of AHF as more than 90% of patients attend an ED for symptom consultation [4]. Following ED patient assessment, hospitalization occurs in the majority of cases. However, depending on the country and/or health care system, between 16 and 36% of patients with an ED diagnosis of AHF are exclusively managed in the ED by treatment initiation or optimization and then discharged home without hospitalization [5]. This attitude has sometimes been criticized, as many specialists feel that nearly every patient presenting HF decompensation, independently of the severity of the AHF episode, should be hospitalized. In fact, the current guidelines of the European Society of Cardiology do not provide any advice or directions for ambulatory management of AHF, and all recommendations are limited to the assumption that the patient will be hospitalized [6].

In the case of ED HF patients with a less severe decompensation, it has been suggested that short stay units (SSU) would be a good alternative to hospitalization for the management of a subset of patients [7–9]. SSU have been specifically designed for hospitalization of patients presenting decompensation of chronic conditions that do not need further investigations and with a predicted length of stay (LOS) not surpassing 4 days in most cases [10]. In fact, in many series AHF constitutes one of the leading causes of SSU admission [11–15]. Nonetheless, in hospitals with an SSU, patients who would otherwise be managed in an outpatient setting could be managed in an SSU to thereby provide more controlled treatment of AHF and improve patient outcomes. This would, however, be more costly compared outpatient management. Comparisons of these two strategies of patient management are limited [8]. The aim of this study was to compare the outcomes of HF patients directly discharged from the ED with comparable patients admitted to a SSU.

## Methods

### Setting

The present study is a subanalysis of the EAHFE Registry. The EAHFE Registry was initiated in 2007 and every 2–3 years carries out a 1–2-month recruitment period of all consecutive patients diagnosed with AHF in Spanish EDs participating in the project. To date, 6 recruitment phases (in 2007, 2009, 2011, 2014, 2016 and 2018) have been performed, with the participation of 45 EDs from community and university hospitals across Spain (representing about 15% of the Spanish public health care system hospitals), enrolling a total of 18,370 AHF patients. Details of patient inclusion have been extensively reported elsewhere [1, 16, 17]. As a retrospective chart review, the EAHFE Registry does not include any planned intervention, and the management of patients is entirely based on the attending ED physician decisions. The only exclusion criteria for inclusion is the development of AHF during ST-elevation myocardial infarction (STEMI), as many of these patients are immediately taken to the cath lab for revascularization, bypassing the ED.

### Study design and variables recorded

The present analysis was limited to patients enrolled in the EAHFE Registry database by the 17 hospitals that had an SSU at the time of patient recruitment. To be included in this analysis, patients had to have been directly discharged from the ED (direct discharge group, DD-G) or hospitalized and managed in the SSU after ED diagnosis and management (SSU group, SSU-G). Patients initially hospitalized in the SSU and then transferred to other hospital departments before final discharge remained in the SSU group. Conversely, patients initially hospitalized in other hospital departments were excluded. All the SSU included in the present study are run by emergency physicians (the majority) or by internal medicine specialists, and it does not include any SSU run by cardiologists. It is a common criterion of the Spanish SSUs that they should admit patients with chronic diseases having a decompensation and not needing any additional investigations aside of follow-up basic laboratory or radiological tests, or patients with an acute disease who is expected to be sent to home after no than 72–96 h (18′, 18′′). Otherwise, patients are hospitalized in conventional wards.

Twenty-eight independent variables were collected. These included demographic data (age, sex), comorbidities (hypertension, diabetes mellitus, coronary artery disease, atrial fibrillation, chronic kidney disease, heart valve disease, chronic obstructive pulmonary disease, dementia, neoplasia, cerebrovascular disease, peripheral artery disease and previous episodes of AHF), chronic treatments (diuretics, beta-blockers, renin-angiotensin system inhibitors, mineral corticosteroid-receptor blockers), baseline status (New York Health Association [NYHA] class, left ventricular ejection fraction, Barthel index), precipitating factors of AHF (infection, tachyarrhythmia, anaemia, hypertensive emergency, dietetic-therapeutic transgression, acute coronary syndrome) and severity of the AHF episode assessed with the MEESSI score. The MEESSI score is calculated from 13 variables recorded during the first patient assessment in the ED (in order of importance: Barthel index, systolic blood pressure, age, NT-proBNP, potassium, troponin, NYHA class, respiratory rate, low output symptoms, oxygen saturation, concurrent acute coronary syndrome, left ventricular hypertrophy in the electrocardiogram and creatinine) [18]. Several studies have demonstrated that the MEESSI score adequately stratifies the risk of death during the following 30 days after ED presentation [18–20].

### Outcomes

We defined two co-primary endpoints that include 30-year all-cause mortality and the 30-day post-discharge combined adverse event. The latter was constituted by all-cause death, hospitalization due to AHF, or ED revisit due to AHF, whichever happened first, and included hospitalizations and ED revisits to any hospital (i.e. they were not limited to the hospital where patients was attended during the index episode). The starting point for the 30-day mortality endpoint was the date of ED consultation, while for the 30-day post-discharge adverse event the starting point was the day of patient discharge after the AHF index event, irrespective of whether discharge was made directly from the ED or after hospitalization in a SSU. For this latter endpoint, patients dying during the index AHF episode (i.e. in-hospital mortality) were not included in the analysis. Vital status, hospitalizations, and ED revisits were ascertained by consultation of medical records, which are electronically accessible in nearly all Spanish hospitals, and by contacting patients or relatives through phone calls when no clear data was present in the clinical history or access was not possible. Follow-up was performed between 30 and 60 days after the index episode. Death was also verified through the Spanish public health insurance database that covers > 99% of the Spanish population, as every patient death is immediately withdrawn from the database at the exact time point that death occurs is reported. Event adjudication was performed at a local level by the principal investigator of each hospital, without external review.

### Statistical analysis

Continuous variables are expressed as median and interquartile range (IQR), and categorical variables as absolute values and percentages. Comparison between groups was carried out using Mann–Whitney rank sums test and the Chi square test (or Fisher exact test, if indicated), respectively. Co-primary outcomes were explored using survival tables and Kaplan–Meier curves, and comparison between curves was made using the log-rank test. Unadjusted and adjusted associations of direct ED discharge and outcomes were calculated using Cox regression models and expressed as hazard ratios (HR) with 95% confidence interval (CI). Adjustment was performed by including the 28 previously described independent variables as covariates. To limit loss of patients in adjusted comparisons, the adjusted analysis was repeated after replacement of missing values using the multiple imputation technique provided by SPSS, generating 10 datasets in which there were no missing values among all the variables included in the adjustment. Mersene’s twister was used as a pseudorandom number generator and 2,000,000 were used as seed. Using this latter adjusted model, we also carried out a subgroup analysis based on sex (female/male), age (< 80 or ≥ 80 years), period of patient inclusion (2009–2014 or 2016–2019), precipitating factor (cardiovascular or not; infection or not), and severity of decompensation based on MEESSI score (low-intermediate risk or high-very high risk), and first-degree interaction for these factors was calculated. Finally, we compared outcomes in a propensity score (PS) matched cohort of paired patients. The PS was calculated by logistic regression (including all 28 independent variables) and determined the probability that patients would be hospitalized in a SSU. Matching was performed following the nearest neighbour matching technique, using the standard deviation of the logit of the PS multiplied by 0.2 as calliper (which resulted in 0.04) for 1:1 patient matching [21].

Statistical significance was defined as a *p* value < 0.05, or if the 95%CI of the HR excluded the value 1. Since this was an exploratory study, a pre-hoc sample size calculation was not made. All calculations were made using SPSS v24.0 software (IBM, Armonk, NY, USA), with FUZZY and PSM Python-based extensions to run PS matching.

### Ethics

The EAHFE Registry protocol was approved by a central Ethics Committee at the Hospital Universitario Central de Asturias (Oviedo, Spain) with the reference numbers 49/2010, 69/2011, 166/13, 160/15 and 205/17. Due to the non-interventional design of the registry, Spanish legislation allows central Ethical Committee approval, accompanied by notification to the local Ethical Committees. All participating patients gave informed consent to be included in the registry and to be contacted for follow-up. The present study was carried out in strict compliance with the principles of the Declaration of Helsinki.

## Results

Of the 18,370 patients included in the EAHFE Registry, 12,117 (66.0%) were from 17 EDs with a SSU. Of these, 2358 (19%) were discharged home from the ED and made up the DD-G, and 2003 (17%) were hospitalized in an SSU, and formed the SSU-G. The overall median (IQR) age was 82 (76–87) years, and 58% were women.

Bivariate analysis comparing the DD-G and SSU-G cohorts showed differences in 17 of the 28 variables (Table 1). DD-G patients were younger, more frequently male, with fewer comorbidities, were less often on chronic treatment with diuretics, and had a better baseline NYHA class and Barthel index. DD-G patients less frequently presented with infection, tachyarrhythmia or hypertensive crisis as precipitants, and their severity of decompensation was lower (Table 1).

**Table 1:** Characteristic of patients with acute heart failure discharged directly home from the emergency department (ED) compared to those admitted to the short-stay unit (SSU)

	All patients			
	Patients with valid values %	ED direct discharge *N* = 2,358 *n* (%)	SSU admission *N* = 2,003 *n* (%)	p
Demographic data				
Age (years) [median (IQR)]	100	81 (74–86)	84 (79–89)	** < 0.001**
Female sex	99.8	1307 (55.6)	1237 (61.9)	** < 0.001**
Comorbidities				
Arterial hypertension	99.7	1924 (81.9)	1740 (87.0)	** < 0.001**
Previous heart failure	99.7	1267 (55.5)	1338 (69.1)	** < 0.001**
Atrial fibrillation	99.7	1141 (48.6)	1140 (57.0)	** < 0.001**
Diabetes mellitus	99.7	917 (39.1)	839 (42.0)	0.051
Ischaemic cardiomyopathy	99.7	620 (26.4)	544 (27.2)	0.555
Valve disease	99.7	537 (22.9)	506 (25.3)	0.060
Chronic renal disease (creatinine > 2 mg/dL)	99.7	527 (22.4)	578 (28.9)	** < 0.001**
Chronic obstructive pulmonary disease	99.6	475 (20.2)	466 (23.3)	**0.013**
Neoplasia	91.9	326 (15.0)	238 (13.0)	0.065
Cerebrovascular disease	99.7	252 (10.7)	275 (13.8)	**0.002**
Peripheral artery disease	99.6	175 (7.5)	185 (9.3)	**0.032**
Dementia	91.9	143 (6.6)	216 (11.8)	** < 0.001**
Chronic treatment				
Diuretics	99.4	1698 (72.5)	1561 (78.3)	** < 0.001**
ACEIs or ARA-II	99.4	1363 (58.2)	1128 (56.6)	0.287
Betablockers	99.4	1141 (48.7)	916 (46.0)	0.068
Aldosterone receptor antagonist	99.4	376 (16.1)	337 (16.9)	0.449
Functional capacity				
NYHA class	95.5			** < 0.001**
I		643 (28.8)	397 (20.6)	
II		1176 (52.7)	1104 (57.2)	
III		396 (17.3)	401 (20.8)	
IV		28 (1.3)	28 (1.5)	
LVEF (%) [median (IQR)]	56.2	55 (44–63)	55 (46–63)	0.360
Barthel index (points) [median (IQR)]	90.2	95 (75–100)	85 (65–100)	** < 0.001**
Precipitating factor				
Infection	88.9	535 (25.7)	699 (39.0)	** < 0.001**
Rapid atrial fibrillation	88.9	286 (13.7)	288 (16.1)	**0.041**
Anaemia	89.0	124 (5.9)	108 (6.0)	0.927
Non-adherence to pharmacological or dietetic treatment	89.0	112 (5.4)	84 (4.7)	0.326
Hypertensive crisis	89.0	94 (4.5)	119 (6.6)	**0.004**
Acute coronary syndrome (either angina or non-STEMI)	98.9	14 (0.6)	22 (1.1)	0.072
Severity of decompensation episode				
MEESSI scale**	57.4			** < 0.001**
Low risk		734 (60.6)	515 (39.8)	
Intermediate risk		394 (32.5)	541 (41.8)	
High risk		44 (3.6)	124 (9.6)	
Very high risk		40 (3.3)	113 (8.7)	

Bold values indicate *p* < 0.05

^*^Propensity score for short-stay unit hospitalization was calculated by multiple logistic regression using all the variables included in the table as covariates (demographic data, comorbidities, chronic treatment, functional capacity, precipitating factors and severity of decompensation episode)

^**^The severity of the episode was estimated with the MEESSI scale which classifies the risk of death of a patient with left cardiac insufficiency in the 30 days following presentation to the emergency department based on 13 variables obtained at arrival to the emergency department: age, Barthel index, NYHA respiratory class, systolic blood pressure, respiratory frequency, oxygen saturation, signs of low cardiac output, episode triggered by an acute coronary syndrome, left ventricular hypertrophy in the ECG and NT-proBNP, troponin, creatinine and potassium values

*IQR* interquartile range, *NYHA* New York Heart Association, *ACEI* angiotensin-converting enzyme Inhibitors, *ARAII* angiotensin receptor antagonists-II, *LVEF* left ventricular ejection fraction, *STEMI* ST-elevation myocardial infarction

**Fig. 1:**
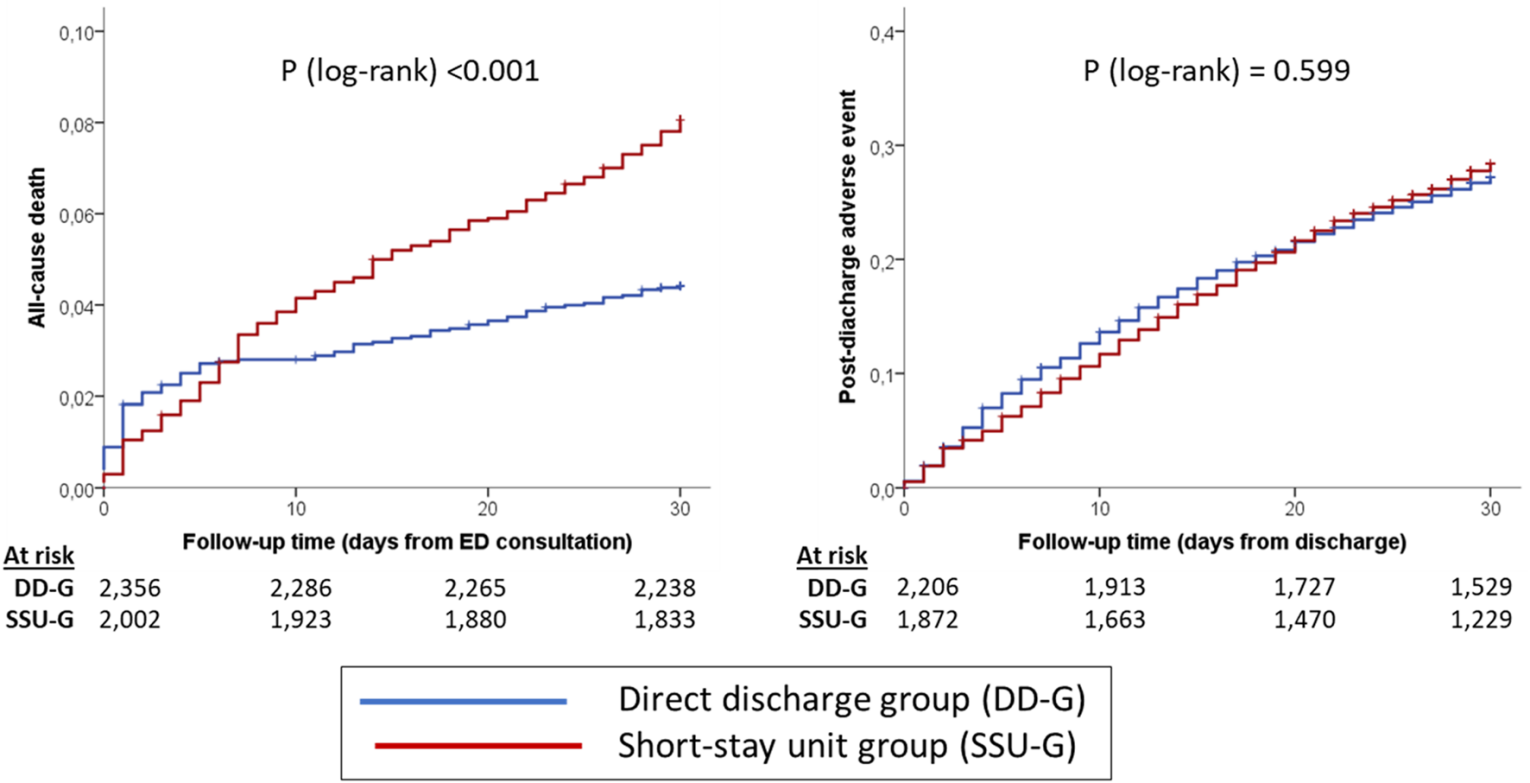
Kaplan-Meier curves depicting the cumulative incidence of death (left) and post-discharge adverse event (right) in the whole cohort of patients included in the present study

**Fig. 2:**
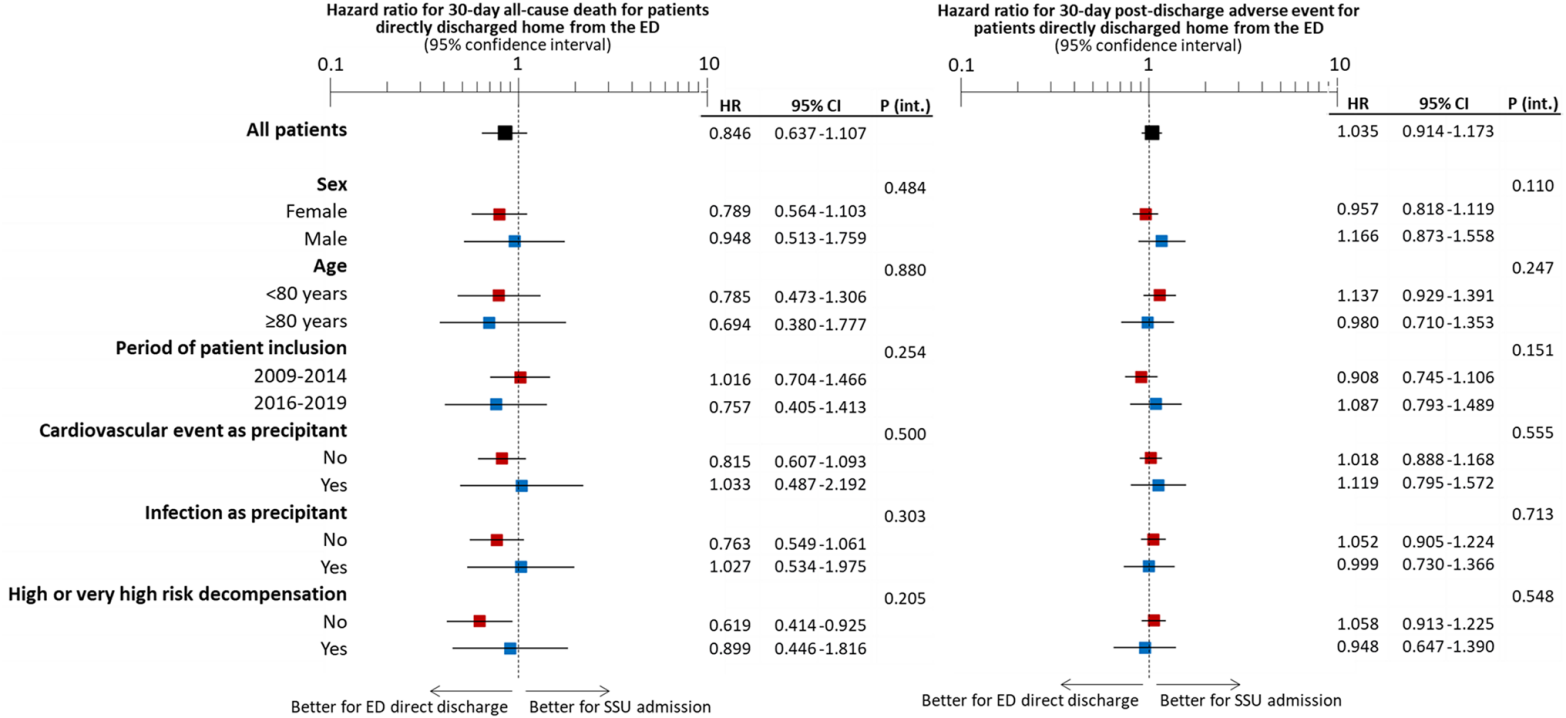
Subgroup analysis of the association between patient disposition (direct discharge from the emergency department or admission to short stay unit) and risk of 30-day mortality (left) and 30-day post-discharge adverse event (right) in the adjusted model (by the 28 independent variables) using multiple imputation

The cumulative incidence of death at 30 days was 4.4% in the DD-G and 8.1% in the SSU-G (Fig. 1, *p* < 0.001). This difference disappeared after adjustment for differences between DD-G and SSU-G, either in adjusted models with or without multiple imputation (Table 2). The median LOS before final discharge of patients in the DD-G and SSU-G groups were 0 (IQR = 0–1) and 4 (IQR = 2–6) days, respectively; and there were 51 (2.2%) and 85 (4.2%) patients in each group that died before being discharged from the current index event. For the patients that survived to the index event, the risk of experiencing a combined adverse event 30 days after discharge was 27.2% and 28.4%, respectively (Fig. 1, *p* = 0.599). Adjustments did not uncover any differences between groups in post-discharge adverse events (Table 2). The subgroup analysis based on the six different factors showed a homogeneous association between disposition and outcomes among all subgroups, without significant interaction for any of them (Fig. 2).

**Table 2:** Outcomes of patients discharged directly home compared to admission to a short-stay unit

	Direct discharge	SSU admission	Hazard ratio (95% CI)
30-day all-cause mortality			
Unadjusted analysis	104 (4.4)	161 (8.1)	**0.544 (0.425–0.697)**
Adjusted analysis, without multiple imputation	–	–	1.024 (0.589–1.783)
Adjusted analysis, with multiple imputation	–	–	0.846 (0.637–1.107)
Propensity score matched sample	89 (5.0)	113 (7.0)	0.791 (0.600–1.045)
30-day post-discharge adverse event			
Unadjusted analysis	595 (27.2)	525 (28.4)	0.969 (0.862–1.090)
Adjusted analysis, without multiple imputation	–	–	0.934 (0.742–1.176)
Adjusted analysis, with multiple imputation	–	–	1.035 (0.914–1.173)
Propensity score matched sample	416 (27.8)	418 (27.8)	1.018 (0.889–1.166)

Bold value indicates *p* < 0.05

**Fig. 3:**
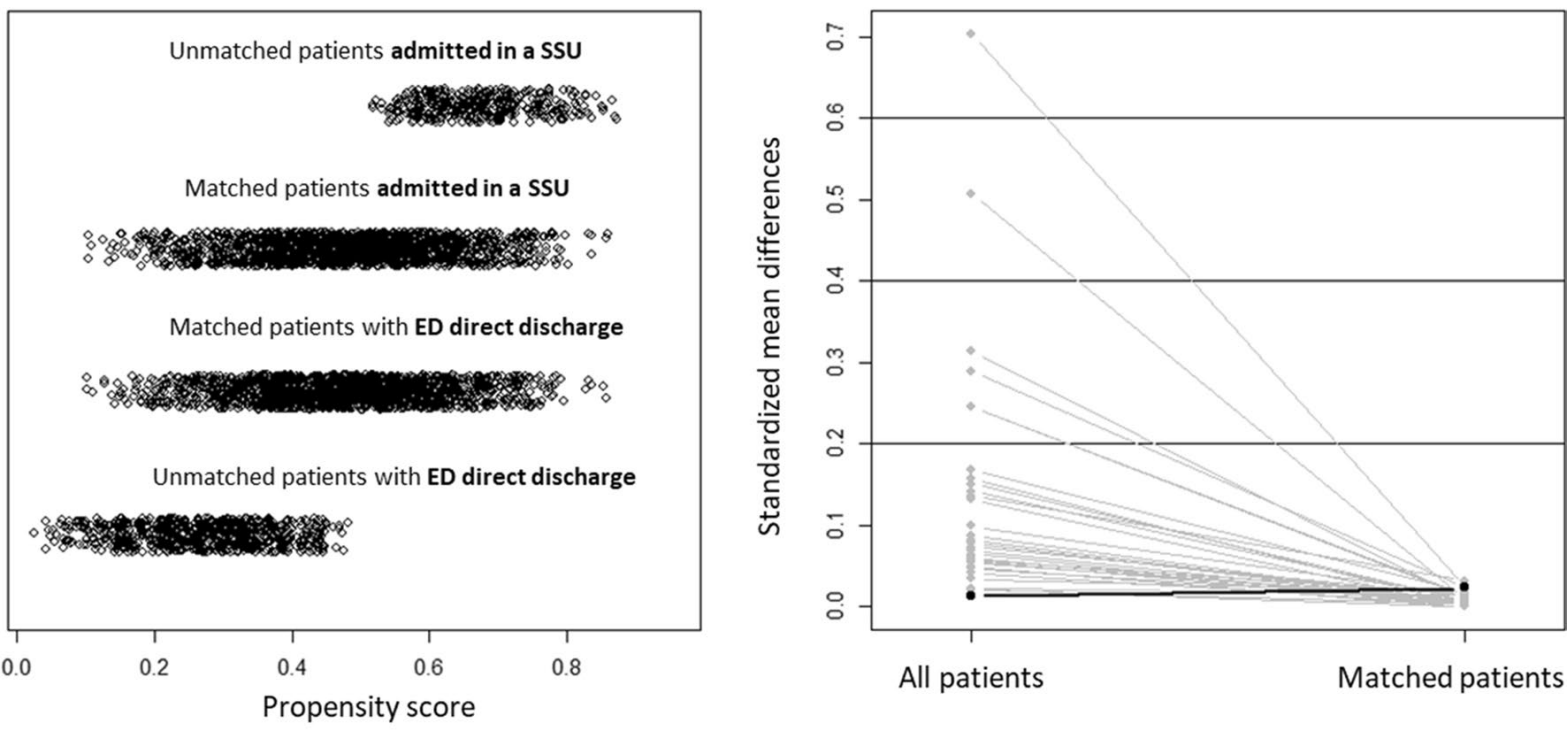
Distribution of propensity score for short-stay unit admission based on 28 independent variables related to baseline status and severity of decompensation in matched and unmatched patients (left) and standardized mean differences for these 28 variables in all and matched patients (right)

**Fig. 4:**
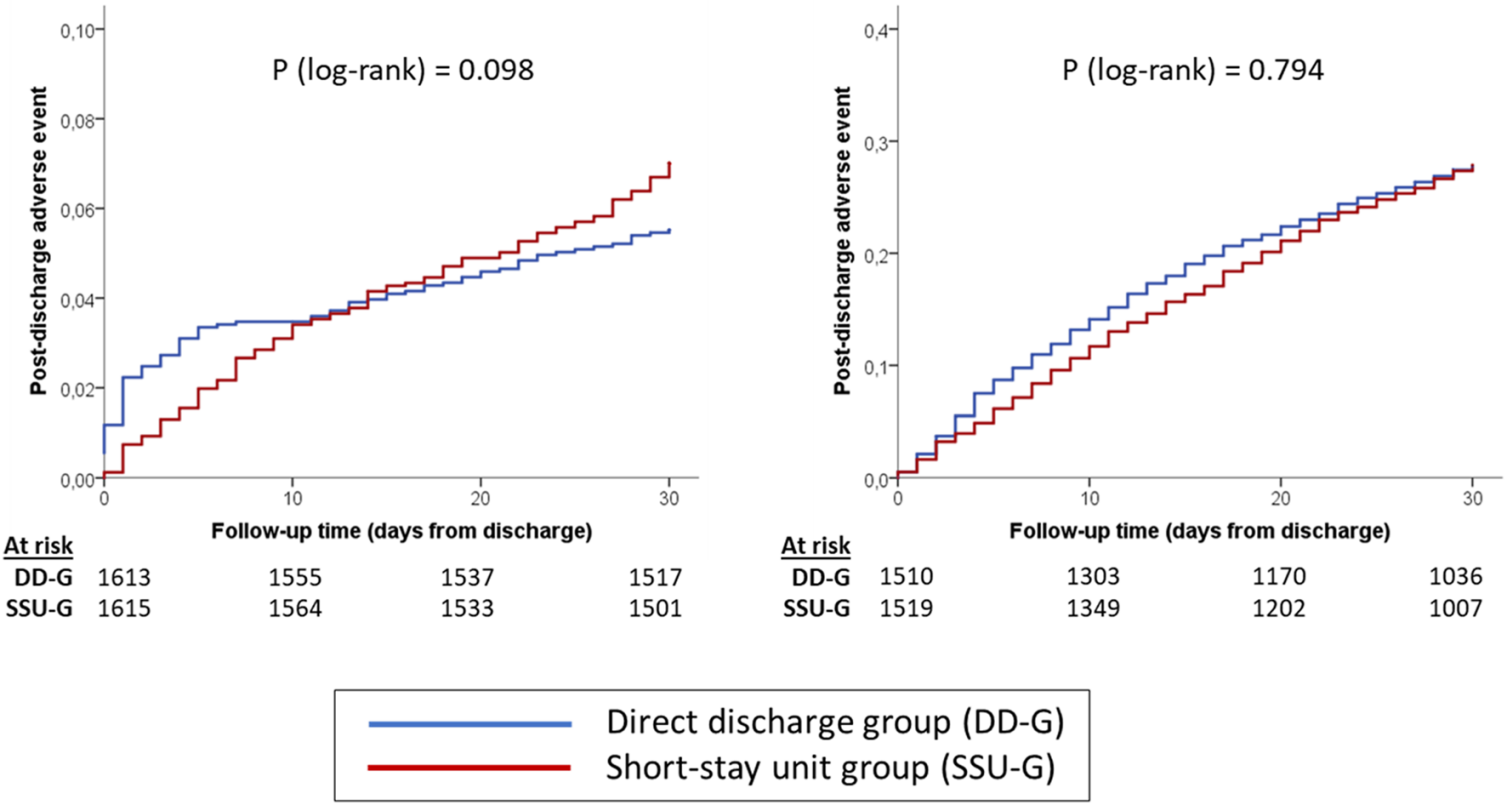
Kaplan-Meier curves depicting the cumulative incidence of death (left) and post-discharge adverse event (right) in the sample of 1615 pairs of patients matched by the propensity score of being hospitalized in the short-stay unit according to 28 independent variables

After PS matching, we identified 1615 pairs of patients from the DD-G and SSU-G with similar probabilities of SSU hospitalization. The patients of the DD-G selected for PS analysis had a higher PS than unselected patients, while the selected patients of the SSU-G had a lower PS than unselected patients (Fig. 3). For all variables, absolute standardized mean differences between both groups used in the propensity score analysis were below 0.1 (Fig. 3). When compared for outcomes, the 30-day cumulative incidence of death was 5.5% in the DD-G and 7.0% in the SSU-G (*p* = 0.098), with a HR for the DD-G of 0.791 (95% CI 0.600–1.045); Table 2, Fig. 4). The median LOS before final discharge of patients in the DD-G and SSU-G were 0 days (IQR = 0–1) and 4 days (IQR = 2–5), respectively. The 30-day cumulative incidence of post-discharge adverse event was 27.8% and 27.8%, respectively (*p* = 0.794), with a HR for the DD-G of 1.018 (95% CI of 0.889–1.166; Table 2, Fig. 4).

## Discussion

In the present study, we found no differences in short-term outcomes between matched patients directly discharged home from the ED vs. those admitted to an SSU. Our results could support a more efficient use of hospital resources in some patients with AHF who are currently hospitalized could be directly discharged home. Nonetheless, it is important to highlight that our finding does not apply to all patients admitted to an SSU, but rather only to those with better baseline status and less severe decompensations. For better identification of this subgroup of patients, risk stratification is a key tool for ED decision-making, as it can help to better discriminate patients who would most benefit from ambulatory management from those really needing hospitalization.

Indeed, the percentage of hospitalizations of patients with AHF attending the ED varies widely worldwide, with higher rates in the US (around 85%) and lower rates in Canada (around 65%) [5]. Differences in health care system characteristics may explain these differences, and may be the consequence of the availability of hospital beds and other resources in each individual centre, as well as the existence of a solid primary care network for patient follow-up. In comparison, in the Spanish hospitals participating in the present study, the rate for hospitalization was 81%, with 17% being admitted to a SSU. Therefore, if part of the 17% of SSU patients were discharged, the overall rate of hospitalization would easily decrease. This shift of selected patients from SSU hospitalization to direct discharge should not necessarily affect short-term outcomes if a proper selection is performed before decision-making. In our series, 27% and 28% of combined adverse events were observed in the DD-G in unadjusted and PS matched cohorts, being lower than the 37.2% observed in the Canadian series [22] and close to the 20–30% adverse event rate in the US [23]. Nonetheless, this is still far from the standard of less of 20% of 30-day ED revisit or hospitalization after ED discharge of patients with AHF proposed by an international expert consensus [5] and, accordingly, caution has to be taken before changing current clinical practice in any particular ED.

One of the main criticisms of the SSU is that its availability may increase the rate of overall admissions [24], which, to some extent, our data confirm. A previous study by our group found that the availability of an SSU was associated with an 8.9% (95%CI 6.5–11.4%) increase in the rate of hospitalizations of HF patients from the ED [25]. However, the presence of a SSU was also associated with a reduction of 10.3% (95%CI  − 16.9% to − 3.7%) in the rate of 30-day ED readmissions following patient discharge, as well as a reduction of 2.2 days (95%CI  – 2.7 to − 1.7) in the overall length of hospitalization. Therefore, the availability of an SSU provides ED physicians with a resource for adequately managing patients who require short-term surveillance vs. those with a more complex baseline situation or more severe decompensation that requires admission and can limit the number of inappropriate discharges with a high risk of repeated ED visits [26]. Previous studies have shown that around 40% of ED AHF revisits following direct discharge are due to non-cardiac problems and, in most patients, these problems were already present in the first evaluation in the ED. Short hospitalization could Likely help to reduce these revisits which, in turn, would improve patient quality of life and reduce health care costs [23].

We want to emphasize that our results do not question the usefulness of a SSU, as they constitute a good tool in the ED for adjusting the necessities of patient admission and in-hospital bed availability [27]. SSUs appeared in the 1980s as an alternative to an increasing demand for hospitalization not covered by conventional hospital wards [8, 28–30]. The efficiency of the SSU model lies in the correct selection of patients to be sent to a SSU. All hospitals with an SSU should have pre-defined illnesses and conditions that can be managed in SSU. It is especially important that patients sent to a SSU after ED care are in a stable clinical and hemodynamic condition at the time of care transition [9–12]. The SSU will thus allow better patient transition to ambulatory care, especially in fragile or dependent patients, who make up a large number of AHF patients and are more likely to have a worse prognosis in the setting of decompensation [31–33]. Moreover, in this group of patients, recommendations and health education are useful for short- and long-term outcome improvement. Finally, short hospitalization could help to achieve more complete decongestion, as well as detect some treatable comorbidities such as iron deficiency, and identify specific triggers of the AHF episode, for starting or titrating some disease-modifying drugs, and ensuring proper patient drug compliance and self-care [25, 34–36]. Admission to an SSU can help to introduce and facilitate all these strategies.

### Limitations

This study has several limitations. First, as in every observational study, causal relationships cannot be inferred and results must be considered hypothesis generating. Additionally, the potential for bias by indication must be considered; i.e. some reasons for sending patients home or to an SSU were not collected as independent variables and, thus, could not be included in the statistical adjustment. Second, there was no sample size calculation, and the lack of statistical significance in some comparisons may have created the potential for beta-error. Third, as the patients came from a nationwide cohort with a universal public health care system, external validation to other systems may require confirmation of their generalizability [37]. Fourth, our study included a high percentage of elderly AHF patients, most with a preserved left ventricular ejection fraction, and in whom frailty and dependence are frequent and as such must be considered when applying our findings to different populations. Fifth, this was real life cohort without any planned intervention, and there could have been differences in physician strategies in treatment and patient disposition. Sixth, the diagnosis of AHF was based on clinical criteria, and the final diagnosis of AHF was not supported by natriuretic peptide or echocardiographic results in all cases. Although these two latter limitations could impose caution in the interpretation of some of our conclusions, this approach makes our findings more generalizable to the real-world emergency medical system and ED practice. Finally, the EAHFE registry only records the department where AHF patients are admitted after ED care, but not further transfers from one department to another before being finally discharged. If this internal transfer happens, it probably denotes a bad patient ED selection for SSU admission in several cases, and this could have partly influenced in our results (as no “pure” SSU patients conform the SSU group).

## Conclusion

With the present results, we can conclude that direct discharge from the ED home in patients diagnosed with AHF is safe and achieves similar short-term outcomes as compared to patients with matched characteristics hospitalized in an SSU. Accordingly, although prospective validation would be helpful, the large size of our cohort suggests that selected patients who are currently admitted to a SSU (those with fewer comorbidities, better status performance, and less severe decompensation) could potentially be considered and safely managed as outpatients, thereby ultimately providing greater efficiency to the health care system. Finally, we also suggest that to adequately identify patients who can be discharged directly from the ED, routine risk stratification is needed before ED decision-making.

## Data Availability

Data available within the article or its supplementary materials.
